# Deciphering Protein Secretion from the Brain to Cerebrospinal Fluid for Biomarker Discovery

**DOI:** 10.1021/acs.jproteome.3c00366

**Published:** 2023-08-22

**Authors:** Katharina Waury, Renske de Wit, Inge M. W. Verberk, Charlotte E. Teunissen, Sanne Abeln

**Affiliations:** † Department of Computer Science, Vrije Universiteit Amsterdam, 1081 HV Amsterdam, The Netherlands; ‡ Neurochemistry Laboratory, Department of Clinical Chemistry, Amsterdam Neuroscience, VU University Medical Center, Amsterdam UMC, 1081 HV Amsterdam, The Netherlands

**Keywords:** brain proteome, cerebrospinal fluid, fluid biomarker, machine learning, protein secretion

## Abstract

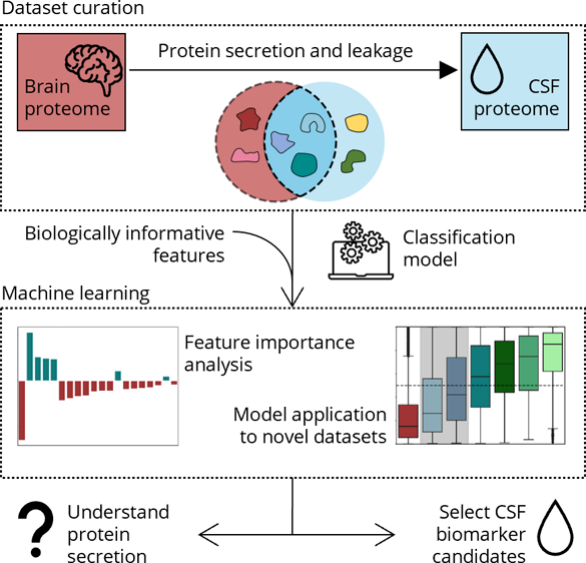

Cerebrospinal fluid (CSF) is an essential matrix for the discovery of neurological disease biomarkers. However, the high dynamic range of protein concentrations in CSF hinders the detection of the least abundant protein biomarkers by untargeted mass spectrometry. It is thus beneficial to gain a deeper understanding of the secretion processes within the brain. Here, we aim to explore if and how the secretion of brain proteins to the CSF can be predicted. By combining a curated CSF proteome and the brain elevated proteome of the Human Protein Atlas, brain proteins were classified as CSF or non-CSF secreted. A machine learning model was trained on a range of sequence-based features to differentiate between CSF and non-CSF groups and effectively predict the brain origin of proteins. The classification model achieves an area under the curve of 0.89 if using high confidence CSF proteins. The most important prediction features include the subcellular localization, signal peptides, and transmembrane regions. The classifier generalized well to the larger brain detected proteome and is able to correctly predict novel CSF proteins identified by affinity proteomics. In addition to elucidating the underlying mechanisms of protein secretion, the trained classification model can support biomarker candidate selection.

## Introduction

Neurological diseases urgently require novel biomarkers to permit patients an early diagnosis, a reliable prognosis, and the appropriate inclusion in clinical trials.^1^ While many biomarker candidates have been identified, very few have reached the end of the biomarker development pipeline and are used in clinical practice.^2^ Additionally, identification of novel biomarkers can support hypothesis generation regarding disease causality and pathogenesis, which for many neurological diseases is still not fully understood.^3^

For the discovery of central nervous system (CNS) fluid biomarkers, cerebrospinal fluid (CSF) is the preferred matrix as it is in close contact with the brain tissue,^4^ yet it can be relatively easily and safely sampled via lumbar puncture.^5^ Nevertheless, several challenges must be considered within CSF biomarker discovery. CSF contains thousands of proteins with a highly dynamic concentration range of an estimated 9 orders of magnitude.^4^ Further, only an estimated 20% of CSF proteins originate from the brain and are able to provide insight into pathological processes of the CNS.^6^

Multiple studies have probed the CSF proteome in healthy humans via discovery proteomics.^7^ These studies are based on “bottom up” mass spectrometry—a hypothesis-free and untargeted method able to identify a high number of unique peptides and subsequently proteins within a sample.^8^ This renders mass spectrometry the most suitable approach to build the proteome of a fluid or tissue of interest.^2^ However, inherent limitations of this technology regarding proteome coverage have to be considered during the biomarker discovery phase.^2,9,10^ While great advances have been made in recent years that allow researchers to overcome many issues, untargeted mass spectrometry workflows still struggle to identify the least abundant proteins in complex biological fluids.^10,11^ Importantly, steps used to improve the detection of the low abundance proteome, e.g., protein depletion and fractionation, have their own drawbacks including decreased workflow reproducibility.^9^ This issue is of high importance as these difficult to detect proteins are often of interest as biomarkers.^4^ It is thus beneficial to exploit alternative approaches to identify low abundance CSF proteins that might constitute novel biomarker candidates.

A way to augment the experimental study of body fluid proteomes for biomarker discovery is in silico protein secretion prediction.^12^ Machine learning-based approaches might help predict proteins with the potential to be secreted to and thus be present in a fluid of interest but that are not detectable by untargeted mass spectrometry. These potential biomarkers might subsequently be measured with more sensitive targeted approaches to confirm the prediction. In addition, machine learning approaches to protein secretion can provide a deeper understanding of the cellular processes.

Multiple protein secretion predictors have been developed,^13^ but only rarely was CSF-specific secretion prediction pursued. A recent prediction model, DeepSec, reported an area under the curve (AUC) of 0.9 demonstrating that the movement of proteins to the CSF is encoded in the protein amino acid sequence.^14^ This study and other recently published methods have applied the increasingly popular approach of deep learning to the task of secretion prediction.^13^ While reporting high model accuracy, biological insight from these models is very limited; the infamous “black box” character of deep learning hinders us to understand how the model makes its decisions. Thus, if gaining biological insights from a prediction model is an additional research aim, use of “shallow” but interpretable models might be more beneficiary.^15^

Accordingly, we were interested in developing a CSF secretion predictor considering two important aspects: 1) By limiting model training to likely CNS-originating proteins, our study focuses on specifically distinguishing between proteins secreted from the brain to CSF and those confined to the brain; 2) We prioritize model interpretability by utilizing an explainable machine learning model and biologically informative features to investigate how the model makes its decisions and to explore the biology behind CSF secretion. Note that while we refer here and throughout this study to protein secretion, this term is meant to include all physiological processes that lead to a brain protein’s presence in CSF.

**Figure 1:**
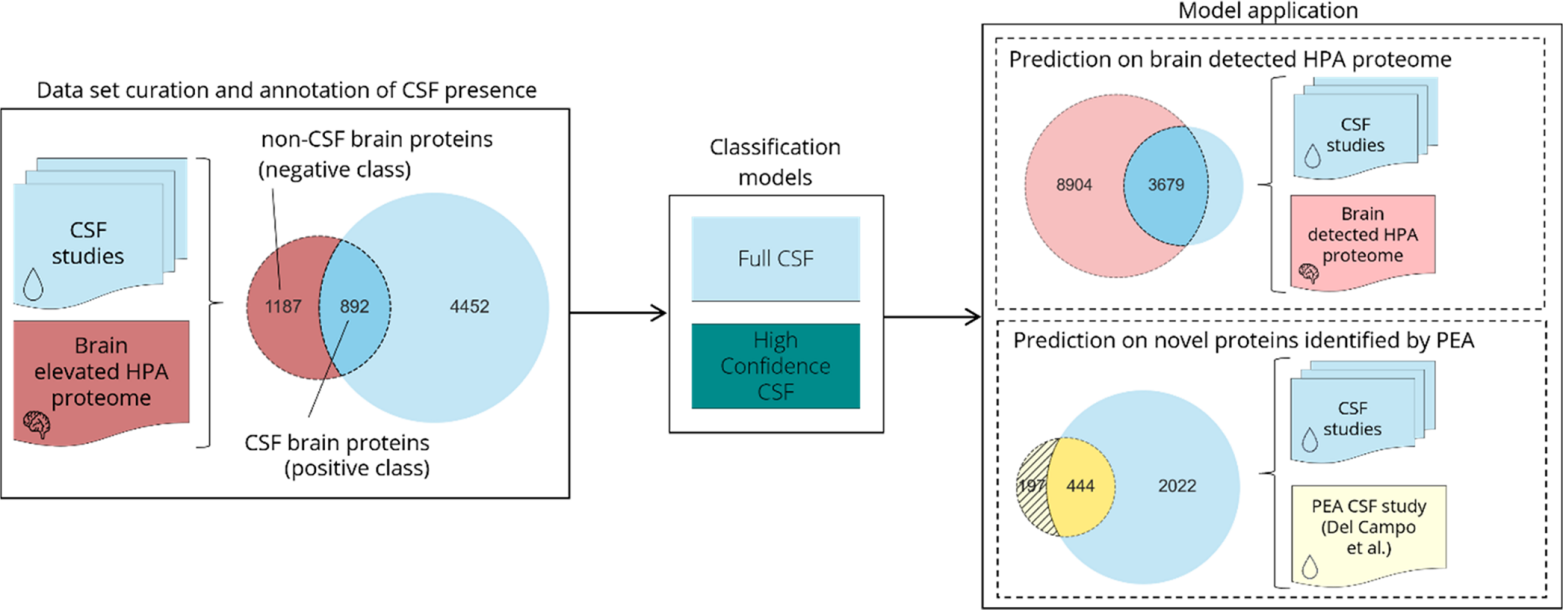
Workflow highlighting the curated data sets and trained classification models. The brain elevated HPA proteome was annotated regarding protein presence in CSF. The resulting data set of CSF and non-CSF brain proteins was used to train the full CSF classification model. By following the same data curation but only including CSF proteins detected in at least half the studies, a high confidence CSF model was trained. The models were applied to two data sets: the brain detected HPA proteome and a set of novel CSF proteins identified by PEA. HPA – Human Protein Atlas; PEA – proximity extension assay.

In this study, we integrated multiple CSF proteomics studies to define and analyze the healthy human CSF proteome. Using this comprehensive CSF proteome, we annotated the brain elevated proteome of the Human Protein Atlas (HPA) regarding CSF presence (Figure 1). We argue that if CSF proteins have an elevated level of brain expression, it is likely that they originated from the brain as opposed to other tissues. Thus, using only the brain elevated proteome, we were able to focus this study on likely CNS-derived proteins. We created numerous sequence-based features for these proteins and trained two machine learning models using CSF proteins of varying stringency (Figure 1). These classification models were able to distinguish between CSF secreted and non-CSF secreted brain proteins, and major differences that elucidate the biological processes leading to the secretion of brain proteins to its proximal fluid were identified. Subsequently, we applied this novel model to the larger brain detected HPA proteome as well as a set of CSF proteins identified by targeted affinity proteomics instead of mass spectrometry (Figure 1). We confirmed that a model trained on healthy CSF proteomics data can be applied to identify disease biomarkers by utilizing Alzheimer’s Disease (AD) studies and known biomarkers as an example.

## Experimental Section

### Data Set Curation

#### Healthy CSF Proteome

To curate a comprehensive proteome of healthy human CSF, previously published CSF proteomics studies were searched and included based on the following requirements: 1) Healthy CSF samples, i.e., without any known diagnoses of neurological disease, were measured; 2) The study was exploratory, not targeted; 3) The study provided a high coverage of the CSF proteome by detecting a minimum of 1000 proteins; 4) The full data set and peptide count were publicly available. These specifications led to the inclusion of six CSF proteomics studies,^7,16−20^ which are summarized in Table 1. The combined list of unique proteins amounted to the full CSF proteome. A high confidence CSF proteome was derived by only including CSF proteins detected in at least half, i.e., three, of the studies. Detailed information about data retrieval and curation is provided in the supplement.

**Table 1:** Included CSF Proteomics Studies to Establish Healthy CSF Proteome

Study name	Reported CSF proteins	Included CSF proteins	CSF sample source	Female:male subject ratio	Median subject age in years	Ref.
Macron2018A	3379	3379	Commercial pool of “normal” CSF	N.A.	N.A.	(7)
Macron2020	3174	3174	Commercial pool of “normal” CSF	N.A.	N.A.	(16)
Zhang2015	3256	2513	Patients undergoing spinal anesthesia	7:7	28 (24–50)	(17)
Guldbrandsen2014	3081	2484	Patients undergoing spinal anesthesia	8:13	61 (19–87)	(18)
Macron2018B	2281	2281	Commercial pool of “normal” CSF	N.A.	N.A.	(19)
Schutzer2010	2630	2067	Healthy volunteers	8:3	28 (24–55)	(20)

#### Alzheimer’s Disease CSF Proteome

A CSF proteome of AD was curated to compare the CSF proteome in health and disease and corroborate that neurological disease does not systematically affect which set of proteins is present in the CSF, but rather alters the abundance of proteins. As AD is a well-researched neurological disease, multiple studies were available that compared the CSF protein abundances of AD patients with those of healthy controls. Three exploratory mass spectrometry studies^21−23^ that respectively identified more than 1000 CSF proteins were identified (Table S1). Further details on the data retrieval can be found in the Supporting Information.

#### Annotated Brain Elevated Proteome

To focus the development of our predictor on protein secretion from the brain to CSF, we limited the included proteins of the healthy CSF proteome to those present in the human brain elevated proteome data set, which was downloaded from the HPA (version 21.0).^24,25^ The HPA also provides information about the average gene expression within the brain. Of the 2709 proteins of the brain elevated HPA proteome, 2546 proteins with a known and unique Uniprot ID were kept. For entries that mapped to more than one Uniprot ID, only the first identifier was retained. Entries with no associated canonical protein sequence or a sequence containing nonstandard amino acids were discarded. Human proteins that have never been detected by any mass spectrometry study according to the ProteomicsDB were excluded as for these proteins it would not be possible to conclude if they are not identified by mass spectrometry studies because of their absence or undetectability.^26,27^ Subsequently, 2079 brain elevated proteins were retained. We annotated this filtered brain elevated HPA proteome as CSF secreted (positive class) and as non-CSF secreted (negative class) if the proteins were present or absent in the CSF proteome (Figure 1). A second annotation used the high confidence CSF protein set to define the positive class correspondingly, while the negative (non-CSF) class was kept the same.

### Gene Ontology Term Enrichment Analysis

Gene ontology (GO) term enrichment analysis was performed using PANTHER (http://www.pantherdb.org/).^28^ The list of 892 CSF proteins was used as the target set, and the filtered brain elevated HPA proteome of 2079 proteins was used as the background set to identify enriched and depleted GO terms in the CSF brain protein set. PANTHER successfully mapped 884 and 2053 proteins of the target and background set, respectively. Fisher’s Exact test was used to test for statistical significance. The false discovery rate was set at .05.

### Feature Generation

We generated a collection of protein (sequence) properties, so-called features, to characterize the proteins in our data set and to train a classifier on. We derived features directly from the canonical amino acid sequence: proportions of each amino acid type, the protein’s physicochemical properties, and instability index.^29^ Additionally, the results of previously published, sequence-based prediction tools for each protein regarding secondary structure and disorder,^30^ signal peptides,^31^ glycosylation sites,^32,33^ subcellular localization,^34^ transmembrane regions,^35^ and glycophosphatidylinositol (GPI)-anchors^36^ were included. The PROSITE protein domain database and the ScanProsite tool were used to identify group enriched sequence pattern motifs and annotate the motif-containing proteins.^37,38^ The use of sequence-based features largely obtained from prediction tools was preferred over curated database annotations to limit the annotation bias in our results.^39^ However, curated protein annotations of ectodomain shedding^40,41^ and extracellular vesicle (EV) association^27^ were taken from previous studies on these properties and investigated to support the interpretation of our model’s findings. Note, however, that these latter annotations were not included in the feature data set used for training the machine learning model. A detailed description of the feature generation process is provided in the Supporting Information.

### Training, Testing, and Interpreting the Predictor

The annotated brain elevated HPA proteome was used to build a machine learning model for the CSF protein secretion prediction. The entire model training workflow described hereinafter was performed both for the full CSF and the high confidence CSF annotations respectively and was carried out using the Python module Scikit-learn.^42^ First, the data was randomly split into a training (80%) and a hold-out test (20%) set. All continuous features were scaled, and the training set was balanced to ensure the same number of proteins in the CSF and non-CSF class. We wished to exclude uninformative features to attain a prediction model as simple as possible. Logistic regression with L1 regularization was implemented, as this model allows the weight of feature coefficients to be set to zero, effectively performing feature selection. To determine the optimal degree of regularization, 10-fold cross-validation of the training set was used to find an optimal regularization parameter C for the model. A C value of 0.5 led to the smallest set of features without a drop in the model performance. The entire training set was subsequently used to produce a trained classifier that is able to distinguish between CSF and non-CSF proteins based on the generated features. The held-back test set provided an estimate how well the trained model would perform on unseen proteins that were not considered during model optimization and training.^43^ The classifier outputs a probability score between 0 and 1 for each protein, with a higher value indicating a stronger probability for this protein to belong to the positive class, i.e., to be secreted from the brain to CSF. The threshold of the model’s predicted probability to be scored as positive (CSF secreted) is set at 0.5. A probability score between 0 and 0.5 indicates that a protein belongs to the negative class and is thus predicted not to be secreted to CSF. Model performance was measured by AUC. Feature importance was studied by extracting the feature weight coefficients of the trained models. A higher absolute coefficient value indicates a higher importance for the model.

### Model Comparison

We compared the predictions on the hold-out test set from our model to DeepSec,^14^ a body fluid-specific protein secretion predictor utilizing neural networks. DeepSec was trained on 6260 CSF proteins collected from previous studies, while the non-CSF proteins were collected from Pfam-defined protein families that are not present in the positive CSF protein set. Note that the training data used by DeepSec is not publicly available; thus, potential overlap between the proteins DeepSec was trained on and the proteins we tested on could not be removed. Proteins for which we wished to compare model performance were submitted to the DeepSec Web server (https://bmbl.bmi.osumc.edu/deepsec/index.php/Home/Index/index.html), and the predicted probability for CSF secretion was obtained for each protein. The model performance was compared by the AUC and sensitivity.

### Application of the Prediction Model

#### Prediction on the Brain Detected Proteome of the Human Protein Atlas

To evaluate how well the model generalized toward all proteins known to be expressed in the brain, features were also generated for the entire brain detected proteome of the HPA (version 21.0).^24,25^ The proteome is composed of 16,507 proteins, of which 16,021 were mapped successfully to a known Uniprot ID. For a fair evaluation of our model’s generalizability, we removed all proteins that are part of the brain elevated HPA proteome from the brain detected HPA proteome, as the model has already seen the brain elevated proteins during training and testing. We removed non-mass spectrometry detectable proteins according to ProteomicsDB;^26^ this left 12,583 proteins in the data set. The brain detected HPA proteome was overlapped with our CSF proteome to count the number of CSF studies in which a protein was found in the same manner as described for the brain elevated HPA proteome. The model was then applied to the brain detected HPA proteome to receive predicted probabilities regarding CSF secretion. For comparison of differences in abundance and expression distribution, data from PaxDB^44^ was used to annotate proteins regarding their average brain abundance; annotations regarding RNA tissue distribution were taken from the HPA.

#### Prediction on CSF Proteins Detected by Affinity Proteomics

We wished to confirm that the trained classifier is able to identify CSF proteins that can only be detected by a targeted approach despite being trained on protein annotations from untargeted mass spectrometry. Successful prediction of such CSF proteins would corroborate that the model truly learned signals of CSF secretion instead of mass spectrometry detectability. A recent study by Del Campo et al.^45^ measured hundreds of CSF proteins in a large cohort of dementia patients by proximity extension assay (PEA). PEA is an antibody-based technology with high sensitivity and medium multiplex capabilities^46^ which has previously been suggested to be used complementarily with untargeted mass spectrometry for biomarker discovery.^47^ We collected the study’s confidently detected CSF proteins and examined our model’s predicted probability for these proteins with a focus on the novel detected CSF proteins, i.e., the proteins not found in the CSF proteome collected from exploratory mass spectrometry studies.

#### Prediction on Established Alzheimer’s Disease Biomarkers

To demonstrate the potential of a machine learning approach to identify the presence of biomarker candidates in CSF, we investigated whether known CSF biomarkers of AD would be predicted as secreted with our approach. A confirmation of established biomarkers would increase the confidence in the model to be able to select novel biomarkers. Seventeen AD biomarkers were collected from recent reviews.^48−50^ We then removed these proteins from our brain elevated proteome, if present, to allow for a fair evaluation of our classifier. The classification model was retrained in the same way as described above. The newly trained model was then used to obtain the predicted probabilities for the AD biomarkers.

## Results

Studies of healthy CSF were combined to define the full and high confidence CSF proteome. We annotated the brain elevated HPA proteome regarding its presence in CSF, created relevant features for these proteins, and trained a machine learning model to correctly classify CSF and non-CSF secreted brain proteins. To understand the processes of protein secretion to CSF, features important for differentiation between the CSF and the non-CSF class were identified. To illustrate our model’s utility, we applied it to the brain detected HPA proteome and an affinity proteomics detected protein set.

### CSF Composition Variability

**Figure 2:**
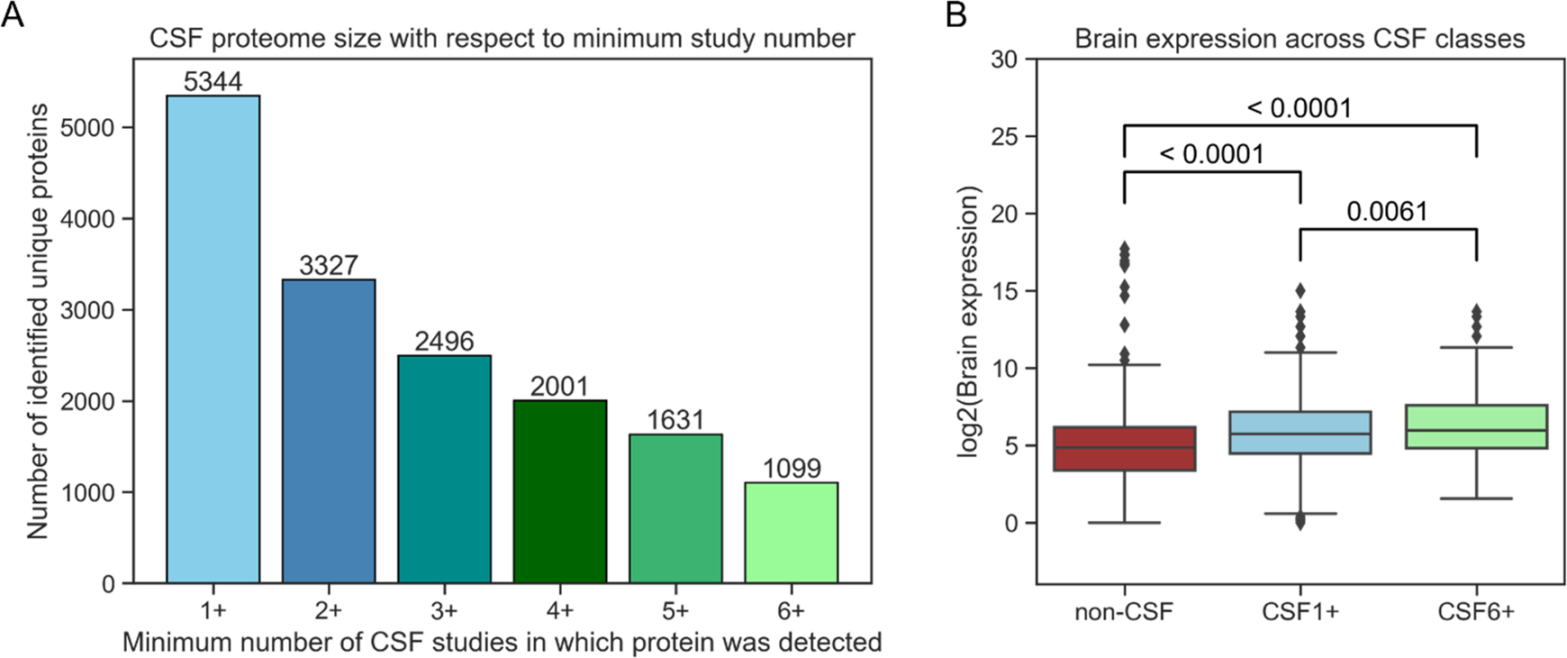
CSF brain proteome. (A) Integration of six different mass spectrometry studies leads to a CSF proteome composed of 5344 unique proteins. Increasing the minimum number of CSF studies that a protein has to be found in leads to smaller but higher confidence CSF proteomes. (B) The average expression of brain elevated proteins according to the HPA is significantly lower in the non-CSF protein group (red) compared with the CSF proteins (CSF1+, light blue). Average expression is even higher in the CSF proteins present in all six studies (CSF6+, light green). HPA – Human Protein Atlas.

From the six included mass spectrometry studies of healthy CSF (summarized in Table 1), 5344 unique proteins were identified in at least one study and thus comprise our full CSF proteome (Figure 2A). While we did not perform a systematic review search, the comprehensiveness of the CSF proteome was affirmed as no single study added more than 10% of novel unique proteins to the full proteome. Note that 2017 proteins were identified in only one of the six studies. Of those, 1125 (21.05% of the entire data set) were identified by a single matched peptide, increasing the risk that these proteins were falsely identified from mass spectrometry experiments. This issue is especially prevalent when combining the results from multiple proteomics studies.^51^ If only high confidence CSF proteins that were detected in three or more studies were included, the fraction of proteins identified by a single peptide drops to 0.07%, starkly limiting the possibility of spurious protein identifications. There is high interstudy variability with overlaps of the identified proteins ranging between 61% and 90% (Figure S1A). Integration of multiple CSF proteomics studies thus enables curation of a more comprehensive and high confidence CSF proteome compared with single study results.

Further, we investigated if a systematic bias in CSF protein presence exists in neuropathology, as it would limit the application of a machine learning model trained on the healthy CSF proteome for the selection of disease biomarker candidates. We compared the composition of the healthy CSF proteome with the AD CSF proteome (Figure S2A). The overlap increases from 87.26% in the respective full CSF sets to 99.06% in the high confidence CSF sets. A pairwise comparison of the relative overlap of identified proteins for each study with the other included healthy and AD CSF studies showed no systematic difference in protein overlap between healthy CSF studies and AD CSF studies (Figure S2B). These results indicate that the CSF protein set is not systematically different in studies of disease, and we thus continued with the healthy CSF proteome for subsequent steps.

### CSF Annotation of the Brain Elevated Proteome

The overlap between brain elevated and CSF proteome increases when including only CSF proteins detected in multiple studies (Figure S1B). This observation indicates that the constant subset of the CSF is increasingly composed of CNS-derived proteins, while the heterogeneity between CSF studies stems from other sources, i.e., plasma-originating proteins.

The HPA provides brain expression levels based on mRNA transcript detection for the brain elevated proteome.^24^ In Figure 2B the expression of different subsets of the brain elevated HPA proteome is shown. The lower average expression of the non-CSF proteins suggests that low abundance may impede detection in mass spectrometry studies. Additionally, the proteins detected in all six studies (CSF6+) show an even higher average expression in comparison to the CSF proteins found in any study (CSF1+). This finding is in line with the known bias of mass spectrometry against lowly expressed proteins.^10^

### Gene Ontology Term Enrichment Analysis

Previous studies have performed a GO term enrichment analysis of their CSF proteome.^7,16^ As the entire human proteome was used as the background set in these studies, CNS-related terms were significantly enriched. Here, we performed a GO enrichment analysis using the brain elevated HPA proteome as the background set to discover over- and under-represented GO terms in the CSF secreted brain protein set. In summary, the enrichment analysis suggests that CSF secreted brain proteins are associated with adhesion function, the membrane, and the extracellular space. Brain-confined proteins are more likely to be located in the nucleus and perform a function related to nucleotide-binding. The full results of the GO term enrichment analysis can be found in Table S2.

### Classification Model Performance

**Figure 3:**
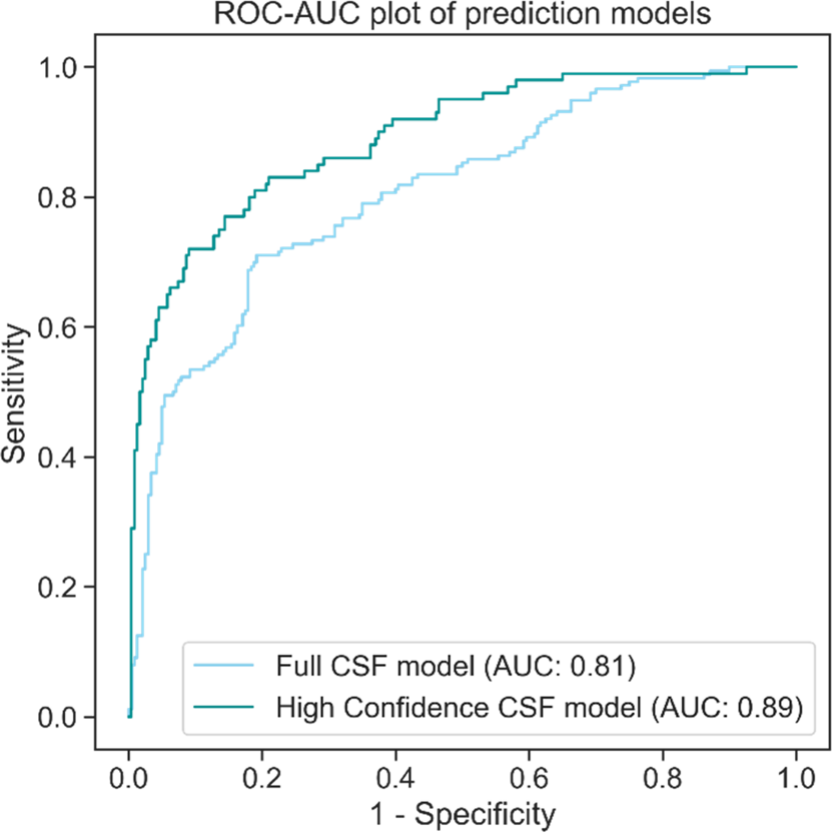
Model performance on the test set. The ROC-AUC plot illustrates how well the two trained prediction models perform on their respective held-back test sets. The high confidence CSF model performs better, indicating that ambiguous proteins were filtered out. AUC – area under the curve; ROC – receiver operating characteristics.

For each protein in our brain elevated HPA proteome an expansive set of features was generated, which are described in detail in the Experimental Section and the Supporting Information. Based on the features, a logistic classifier (also known as a logistic regression model) was trained to distinguish between the positive (CSF secreted brain proteins) and negative classes (non-CSF secreted brain proteins). Evaluation on a held-out test set produced an AUC of 0.81, clearly indicating that there is a discrepancy between the two classes to be learned from the included features (Figure 3).

As stated earlier, the full CSF proteome might contain falsely discovered proteins, because of the large fraction of single-peptide identifications. Wrongly annotated proteins add “noise” to the data, which hinders machine learning models from discerning biologically relevant signals. Additionally, proteins that cannot be detected routinely in CSF and require specific conditions to be detected would not constitute good fluid biomarkers. Thus, we investigated if limiting the model to learn only from high confidence CSF proteins would improve the accuracy. CSF proteins that were detected in only one or two studies were removed from the data all together; thus, the non-CSF secreted class remained unchanged. The AUC increased to 0.89 for the held-out test set using the high confidence CSF model (Figure 3). The stronger discrepancy between non-CSF and CSF proteins indicates that ambiguous proteins were removed when CSF proteins detected in a small number of studies were excluded. Prediction of CSF secretion is evidently possible with our approach, especially for high confidence annotations.

### Model Comparison with DeepSec

The performance of the high confidence CSF model and DeepSec was compared for the 368 proteins used as the hold-out test set (Table 2). Our model performs better on the held-out test set with an AUC of 0.89 compared to an AUC of 0.82 of DeepSec.

**Table 2:** Performance Comparison of the High Confidence CSF Classification Model and DeepSec on the Held-Back Test Set of the High Confidence CSF Trained Model and the Novel Set of CSF Proteins Identified by PEA

Model	High confidence CSF		DeepSec	
Accuracy metric	AUC	Sensitivity	AUC	Sensitivity
Hold-out test set (368 proteins)	0.89	76.00%	0.82	72.00%
PEA CSF proteins (197 proteins)	–	75.13%	–	71.57%

### Analysis of the Most Important Classification Features

**Figure 4:**
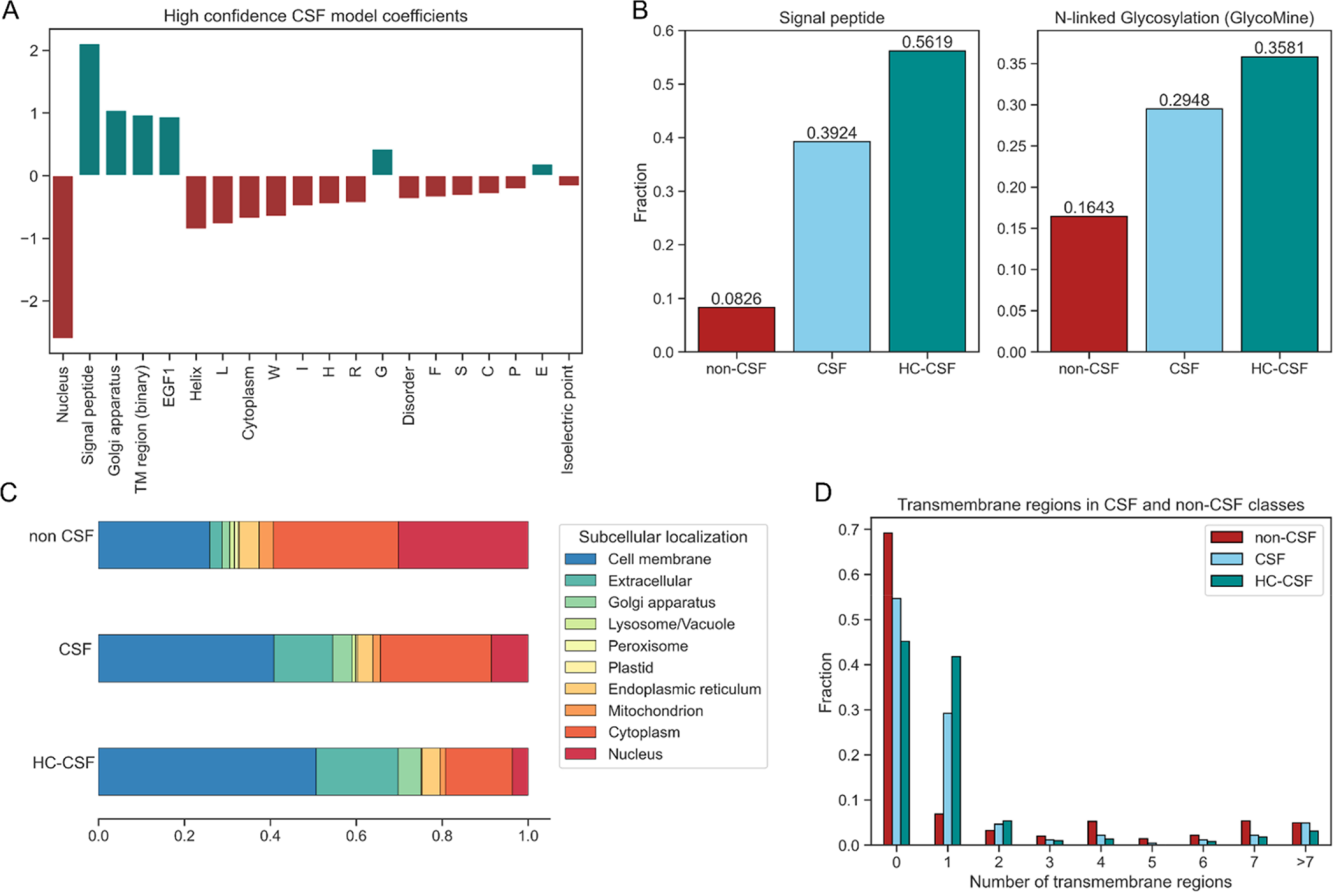
Features most important for classification. (A) The highest absolute feature coefficients of the high confidence CSF model indicate which features are relevant for the model’s decision-making. (B) Features associated with the conventional secretion pathway of the cell, e.g., the presence of a signal peptide and glycosylation sites in the protein sequence, are more common in CSF secreted proteins. (C) The proportions of predicted subcellular localizations show clear differences between the CSF and non-CSF group. (D) While proteins with one predicted transmembrane region are much more likely to be found in the CSF, the opposite is true for proteins with a high number of transmembrane proteins. HC-CSF – high confidence CSF; TM – transmembrane.

One major advantage of a simple classification algorithm is the rather direct readout of a feature’s importance to the model’s decision. In the case of a logistic classifier, the feature importance is related to the learned feature coefficients: a higher absolute coefficient value indicates a higher importance. A positive value indicates correlation with the positive class (CSF secreted), and a negative value indicates correlation with the negative class (non-CSF secreted). The 20 most important features of the high confidence CSF model are shown in Figure 4A.

The presence of a signal peptide within the protein’s sequence is a highly important feature utilized by the high confidence CSF model to identify secreted proteins (Figure 4A). This relevance is expected as it is a cellular indicator to secrete the protein. Glycosylation sites are also found more frequently in CSF secreted proteins (Figure 4B), as glycosylation is part of the conventional secretion pathway involving the endoplasmic reticulum and the Golgi apparatus.^52^

The subcellular localization of a protein, e.g., in the nucleus, the Golgi apparatus, or the extracellular space, is highly important for correct classification of CSF and non-CSF brain proteins. Examining the distribution of predicted subcellular locations illustrates the differences between CSF secreted and non-CSF secreted proteins (Figure 4C). The stronger association of CSF proteins with the cell membrane, the extracellular region, and the Golgi apparatus is evident, as the trend becomes stronger in the high confidence CSF protein set. In contrast, the CSF proteome contains only a small fraction of proteins of the nucleus and the mitochondrion compared to the non-CSF proteome.

Interestingly, the presence of transmembrane regions as a binary feature is positively correlated with CSF secreted proteins, while the number of transmembrane regions shows a negative correlation. To understand this apparent paradox, we investigated the fractions of CSF and non-CSF proteins per number of transmembrane regions. Figure 4D illustrates three observations: 1) Proteins without any transmembrane region are slightly more often retained in the brain (i.e., non-CSF); 2) One transmembrane region is strongly associated with the protein’s presence in CSF; 3) If a protein has many transmembrane regions, correlation with the non-CSF class becomes stronger again. These observations are even more evident in the higher confidence CSF. Although initially it might seem intuitive that proteins with a transmembrane region are restricted to the brain, our findings might be explained by fragments of a membrane protein’s extracellular domain easily leaking into the CSF, by ectodomain shedding, or by membrane association with EVs that can be found in all body fluids. To investigate the latter two circumstances, we utilized data sets on ectodomain shedding and EV-associated proteins and examined their overlap with CSF and non-CSF proteins. Both ectodomain shedding and EV-associated proteins are highly over-represented in CSF secreted proteins, indicating that both processes are relevant for presence of brain proteins in CSF (Figure S3). Note that these annotations were not included in the prediction model, as they cannot be considered sequence-based.

We identified three motifs that are enriched in CSF brain elevated proteins and three motifs that are enriched in non-CSF brain proteins. Associated with the CSF class are the EGF-like domain signature 1, EGF-like domain signature 2, and the Cadherin domain signature. In the non-CSF class, we identified the G protein-coupled receptors family 1 signature, the Zinc finger C2H2 type domain signature, and the “Homeobox” domain signature. While none of these patterns are associated with a great number of proteins, enrichment in their respective protein group is strong and is still observed in the higher stringency data sets (Figure S4). These motifs affirm the properties of CSF and non-CSF brain proteins: EGF domains are found in membrane-associated and extracellular proteins.^53^ Cadherins are known to be glycosylated and involved in cell–cell adhesion processes through their extracellular domain.^54,55^ G protein-coupled receptors are firmly incorporated into the membrane through their seven transmembrane regions.^56^ Both the Zinc finger and “Homeobox” domain are involved in nucleotide binding.^57,58^

### Model Prediction on the Brain Detected Proteome

Machine learning approaches can be utilized to understand the systematic differences between two groups of interest. Ultimately, however, the aim of training a classification model is to apply it to novel data and utilize the predictions to guide future research.

**Figure 5:**
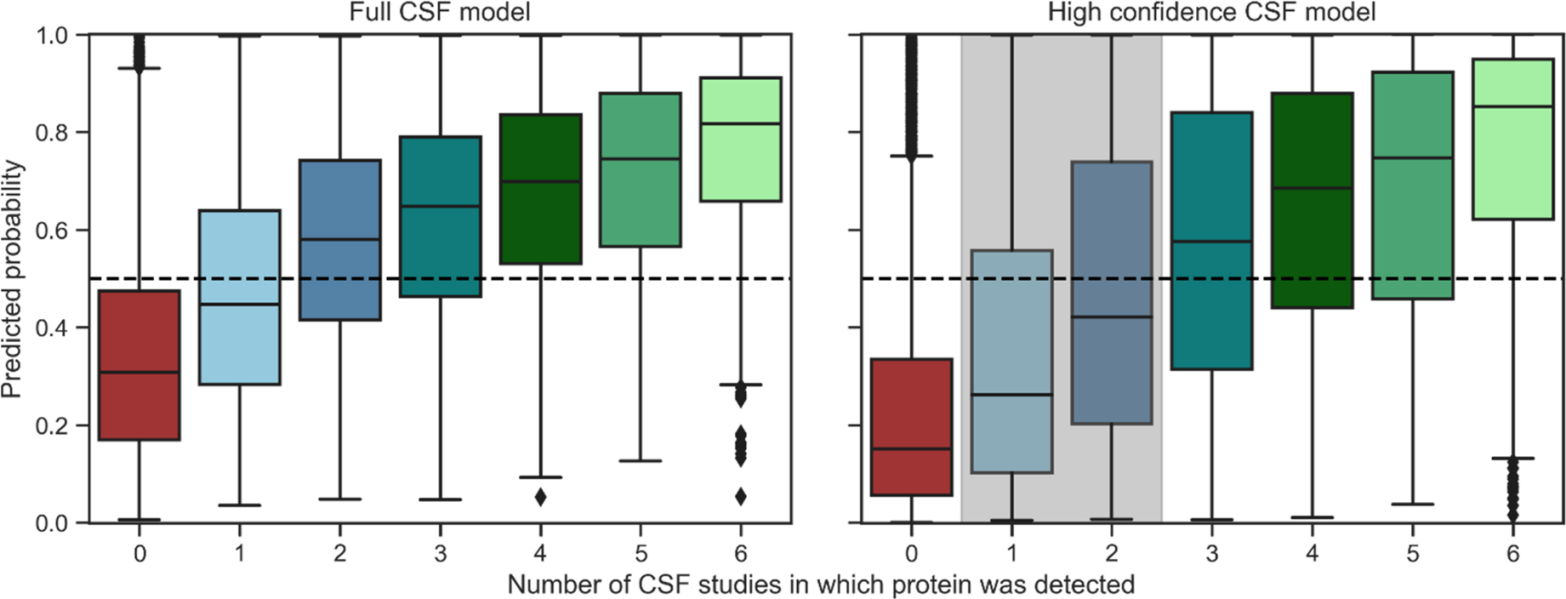
Model performance on the brain detected HPA proteome. Predicted probability of the full CSF and high confidence CSF model on proteins detected in the human brain that have not been utilized for previous training and testing. Proteins with a probability score of >0.5 are predicted as CSF secreted. Both models are most confident about proteins that have been detected in a higher number of CSF studies. Proteins not identified in CSF are consistently predicted as brain confined. The high confidence model predicts a large fraction of ambiguous proteins (marked in gray) as brain-confined. HPA – Human Protein Atlas.

We explored how well our model, which was trained on the brain elevated HPA proteome (2079 proteins), performed on the brain detected HPA proteome (14,662 proteins). Note that while brain detected proteins are expressed in the brain, their origin when detected in CSF is less certain, as these can be expressed highly in other tissue(s) as well. Nevertheless, the model should be able to identify the proteins with the potential to be secreted from the brain to the CSF. To evaluate the model’s performance more fairly, we removed the proteins present in the brain elevated HPA proteome used during training from the brain detected HPA proteome, leading to the model being applied to 12,583 proteins with 3679 of these overlapping with our full CSF proteome (Figure 1). In Figure 5 the predicted probability scores for the brain detected proteome are displayed for the full and the high confidence CSF model, respectively. The CSF proteins were partitioned based on the number of studies of the healthy CSF proteome in which the protein was found. Both models assign a higher probability to CSF proteins than to non-CSF proteins. It is apparent that the models are more confident about the proteins for which the evidence of CSF presence is the strongest, i.e., the proteins that are routinely found in CSF studies. Proteins that have been identified in only one study are more often predicted as non-CSF proteins, again indicating that this subset might contain falsely identified proteins as well as proteins that are inconsistently present in CSF and thus would not constitute favorable biomarker candidates. Noticeably, the probability score for proteins identified in one or two studies drops in the high confidence CSF model (shown in gray in Figure 5). This observation highlights that if ambiguous proteins are not presented to the prediction model during learning, it classifies them as negative. Many of the proteins that were identified in only one or two studies thus show similar properties as non-CSF proteins instead of CSF secreted proteins. This indicates that many of these proteins are indeed false positive identifications in the mass spectrometry experiments, only detectable under ideal conditions, or blood-originating proteins instead of true CSF secreted proteins. The classification model generalized well to this larger brain proteome suggesting its use to guide biomarker selection for proteins that were not part of the brain elevated proteome.

To better understand misclassification of our model, we investigated false negatively predicted proteins, i.e., proteins that were detected in CSF by proteomics studies but with low probability to be CSF secreted according to the prediction model. We were able to retrieve average brain abundance values for 7796 out of the 12,583 proteins from PaxDB; RNA tissue distribution classification from the HPA was available for the full protein set. Comparison of average abundance and RNA tissue distribution suggests that the falsely negative predicted proteins are highly abundant in the brain and expressed widely across all human tissues (Figure S5). Thus, these proteins might have a high chance to reach the CSF (and other body fluids) owing to their high concentration levels and ubiquity, despite not carrying the typical properties of CSF secretion according to the machine learning model.

Of strong interest for CSF biomarker research is the false positive predicted proteins with a high probability score. If a protein has not yet been detected in exploratory mass spectrometry studies and is thus annotated as a non-CSF protein but is confidently predicted to be secreted to CSF by our model, this might indicate that the protein is present in CSF but is not easily detected by mass spectrometry. An obvious reason might be low abundance, which would require ultrasensitive methods to measure this protein in a complex matrix. We provide the probability score of our models for almost every protein in the human proteome including information on its presence in the included CSF studies, the brain detected and the brain elevated HPA proteome (Table S3).

### Prediction on CSF Proteins Detected by Affinity Proteomics

**Figure 6:**
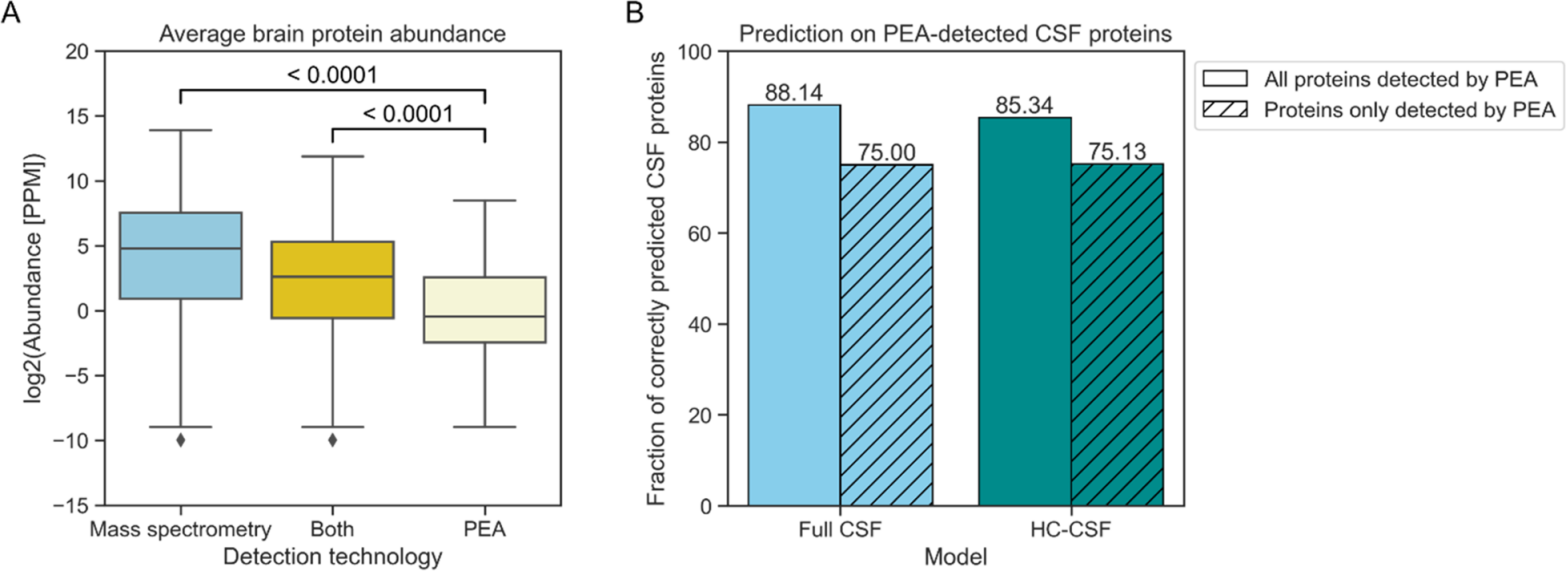
Model performance on proteins identified by affinity proteomics. (A) Proteins detected solely by PEA and not mass spectrometry have a lower average brain abundance according to PaxDB. (B) The classification models perform well on the CSF proteins identified by affinity proteomics, identifying the majority of them. Importantly, the models are able to correctly predict low abundance proteins that are potentially only identifiable by targeted approaches. PEA – proximity extension assay; PPM – parts per million.

As the CSF annotations that the model was trained on solely derived from the results of untargeted mass spectrometry-based proteomics studies, we examined if proteins identified by a targeted affinity proteomics approach, especially those of low abundance, are correctly predicted as well. Del Campo et al. reported 642 proteins that were measured with high confidence in CSF by PEA.^45^ All but a single protein could be matched to a unique Uniprot ID and were retained. While some overlap exists between this PEA set of CSF proteins with the high confidence CSF set curated by mass spectrometry, the PEA-based workflow identified 197 novel CSF proteins (30.73% of the full PEA protein set) that are not part of the high confidence CSF set (Figure 1). Using reported protein abundances of the integrated brain data set of PaxDB, a clear difference in abundance between CSF proteins identified by mass spectrometry and by PEA is found (Figure 6A). PEA thus was able to identify part of the low abundance CSF proteome. The full and high confidence CSF models correctly identify 88.14% and 85.34% of the PEA CSF proteins, respectively (Figure 6B). Importantly, the models still perform well on the subset of novel CSF proteins, as the high confidence CSF model still predicts 75.13% correctly. This performance highlights that the machine learning approach can predict the low abundance CSF proteins not detected by mass spectrometry. Many of the false positive predicted proteins according to the mass spectrometry-based CSF annotations will be actual CSF proteins detectable by targeted approaches. Note that because PEA measures a predefined subset of proteins, no negative protein set can be defined to compare the model prediction with. The performance of DeepSec on the novel PEA-detected subset of CSF proteins was assessed as well (Table 2). DeepSec correctly predicts 71.57% of these CSF proteins.

### Prediction on Established Alzheimer’s Disease Biomarkers

**Figure 7:**
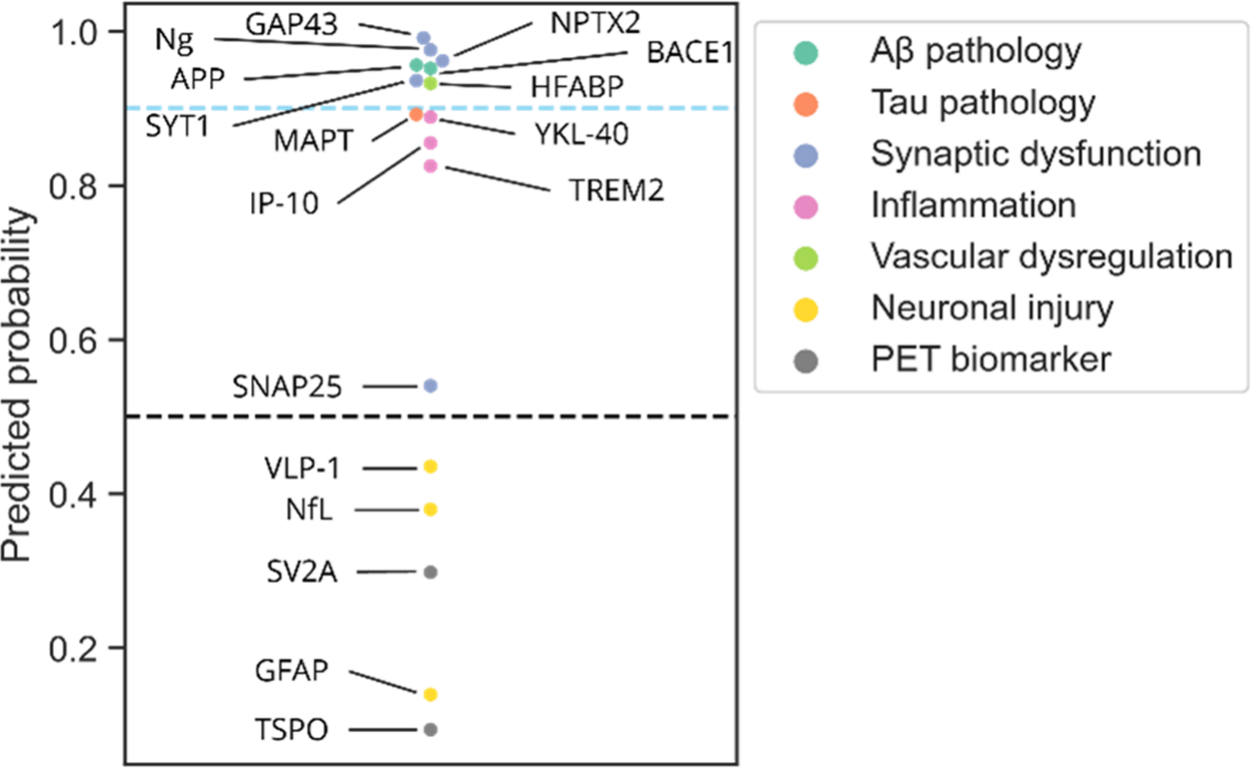
Predicted CSF secretion probability of established biomarkers of Alzheimer’s Disease. A model was trained on the high confidence CSF data set but with a list of 17 AD biomarkers removed. The model correctly predicts 12 out of 15 CSF biomarkers as being secreted to the CSF, many with a very high probability. Colors indicate the process the biomarker is associated with, illustrating that the model struggles to identify biomarkers of neuronal injury as CSF secreted. Two known PET biomarkers are predicted as non-CSF proteins. The prediction corroborates why for these two imaging biomarkers no assay for measurement in CSF is established. AD – Alzheimer’s Disease; NfL – neurofilament light chain; Ng – neurogranin; PET – positron emission tomography.

To illustrate the potential to select biomarker candidates by incorporating machine learning-based predictions regarding protein secretion, we trained a model on the brain elevated HPA proteome annotated in the same manner as described earlier but with a list of 17 AD biomarkers removed before training. This new model was trained using the same features but has not “seen” the AD biomarkers during training, which allows determination of how well it predicts these proteins without having knowledge about their presence in CSF beforehand. We included 15 proteins that have been suggested as fluid CSF biomarkers in AD, as well as two imaging protein biomarkers that are detected in positron emission tomography (PET) scans of the brain. Note that while these two proteins, SV2A and TSPO, are protein biomarkers, they are not necessarily expected to be present in CSF as they are not fluid biomarkers. The predicted secretion scores for these 17 biomarkers are shown in Figure 7. The model predicted the majority of fluid biomarkers as CSF secreted, i.e., with a score of >0.5. Interestingly, annotating the biomarkers regarding the cellular process they reflect illustrates that the three fluid CSF biomarkers not predicted correctly, Neurofilament light chain (NfL), GFAP and VLP-1, are all associated with neuronal injury. This observation indicates that the model will struggle to identify proteins that are present in CSF because of brain cell death. This circumstance is not surprising as apoptosis and disintegration of brain cells would lead to a nonselective movement of proteins to the surrounding fluids. The low secretion probability predicted for the PET biomarkers emphasizes that this model could guide identification of proteins that are recognized to be of interest in brain tissue proteomics studies but are unsuitable to be translated to fluid biomarkers. This use case of established fluid biomarkers of AD confirms the utility to identify candidates based on the model’s probability score. Thus, future studies with the aim to select and validate novel proteins as biomarkers could benefit from the incorporation of the model prediction scores.

## Discussion

To identify urgently needed novel CSF biomarkers, a deeper knowledge of brain protein secretion and leakage processes will be of great help. Interpretable machine learning is a valuable approach to widen our understanding as well as to discover novel biomarker candidates that might be difficult to detect by the conventional workflow, i.e., using “bottom up” proteomics, because of their low abundance in CSF. Utilizing information gained from prediction tools as the one presented here is a rapid, effortless and cost-effective approach to support biomarker candidate identification and selection.^12^

Here, we built a CSF-specific protein secretion prediction model, using a curated CSF proteome data set and the brain elevated HPA proteome, which provides valuable insights for fluid biomarker research. Our predictor is able to distinguish between CSF secreted and non-CSF secreted proteins, with an AUC between 0.81 and 0.89 depending on the stringency used to define the CSF proteome. Our model outperformed the deep learning-based DeepSec predictor on two independent test sets. Interpretation of the model gave insights into the properties that distinguish CSF and non-CSF proteins, including signal peptides and subcellular localization. Finally, we demonstrated the generalizability of this model for the brain detected proteome and for proteins detected by targeted, sensitive technology.

This study confirmed the high heterogeneity between different CSF studies that can be explained by a manifold of factors including differences in biology, sampling, and technology.^4,6,59^ While we only included proteins of CSF samples that were deemed healthy, the true absence of any neurological disease from a CSF sample is difficult to determine. Thus, it was essential to confirm that protein presence in CSF is not systematically different in disease (Figure S2). We provide the largest publicly available collection of healthy CSF proteins, as the integration of several studies provides higher proteome coverage. Note that while the CSF data set reported by DeepSec is larger (6260 proteins), it has not been made publicly available. Wrongly included proteins were less likely to appear in our high confidence CSF set. The positive effect of data filtering on model performance affirmed the importance of a clean data set for successful implementation of machine learning approaches. Correct predictions on a larger brain proteome (Figure 5), as well as proteins identified by a workflow other than untargeted mass spectrometry (Figure 6) indicate that our model generalizes well and can thus be used to characterize new proteins of interest regarding their suitability as a fluid CNS biomarker. The provided model results could be used to exclude unfavorable candidates and to identify very strong candidates before moving to targeted, sensitive technologies to validate these proteins.

While the conventional protein secretion pathway clearly contributes highly to the content of the CNS-derived CSF proteome (Figure 4), our results implicate additional mechanisms, such as ectodomain shedding and association with extracellular vesicles (Figure S3). While membrane-associated proteins might initially not appear as good biomarker candidates, this study strongly suggests otherwise. The results are in line with reports on a few established membrane-associated CSF biomarkers, e.g., the ectodomain shedding protein TREM2.^60^ Adhesion proteins were also identified as a specific group of interest based on GO term and motif enrichment results. Their potential as biomarkers for neurodegenerative disease has already been reported.^61,62^ Our results strongly imply that nucleotide-interacting proteins rarely constitute good fluid CSF biomarkers, which may explain the difficulty to develop fluid biomarker tests for the frontotemporal dementia-related protein TDP-43.^63^

Limitations of our approach and thus of the model’s applicability have to be considered when interpreting results. The use of a simple machine learning approach as well as only sequence-based features might limit the accuracy of the model. It is important to keep in mind that while features derived from other machine learning prediction tools are usually more comprehensive, they might not be as accurate as observations, e.g., from mass spectrometry studies. However, to circumvent annotation bias and lost interpretability,^15,39^ we actively decided against other strategies as the research aim included biological interpretation. Nevertheless, as a deep learning-based predictor did not perform better than our model, the prediction task investigated here might not warrant the use of such sophisticated models because of the limited amount of data available. Still, a drawback of our approach is the extensive feature generation required for the model. While we do provide the probability scores for almost the full human proteome (Table S3), prediction on a novel sequence would require effort beforehand to engineer the features. The predictions for known AD biomarkers (Figure 7) indicate that the model would struggle to identify CSF proteins that reach the fluid because of general cell disintegration during apoptosis. As this process would not be selective for a specific protein group, the model is not able to learn any signal. It is thus important to consider knowledge about the pathological processes associated with the disease of interest when inspecting the predicted probabilities.

A more in-depth analysis on the peptide, instead of protein, level could give insights into the specific proteoforms present in CSF, i.e., specific splicing isoforms or post-translationally modified residues. As biomarkers can be proteoform-specific, with phosphorylated tau presenting one of the hallmark AD biomarkers,^1^ this detailed information would provide further insights. The development of a fragment- or residue-specific predictor of CSF presence would therefore be an interesting way to build on this study.

The biological insights derived from the model can be easily understood by biomarker researchers without the need for strong machine learning domain knowledge. To the best of our knowledge, such an in-depth analysis of the differences between CSF-secreted and brain-confined proteins has not been done previously.

## Conclusions

While biomarker research has been able to make immense progress in recent years because of advances in mass spectrometry-based discovery proteomics, approaches to augment these results should still be pursued. Effort must be put into identifying so far potentially overlooked candidates that could improve the diagnosis and treatment of patients. This study provides one approach to increasing our knowledge of brain-to-CSF protein secretion and can support the search for the next fluid CNS-derived biomarkers for neurological diseases.

## Data Availability

The code and data used in this work are publicly available at https://github.com/kathiwaury/brain-csf-proteomics.

## Supporting Information Available

Supplemental Data: Supporting Information for the Experimental Section;^64,65^ Table S1: Included CSF proteomics studies to establish the Alzheimer’s Disease CSF proteome; Figure S1: Overlap of CSF and brain proteomes; Figure S2: Overlap of CSF proteomics studies in health and Alzheimer’s Disease; Figure S3: Ectodomain shedding and extracellular vesicle (EV) association in CSF and non-CSF brain proteins; Figure S4: Enriched motifs in CSF and non-CSF brain proteins; Figure S5: Differences in brain protein abundance and RNA tissue distribution in false negative predicted brain detected proteins (PDF)
Table S2: Gene ontology term enrichment analysis of CSF and non-CSF brain proteins (XLSX)
Table S3: Predicted probability scores for full human proteome (XLSX)

## ABBREVIATIONS

AD: Alzheimer’s Disease
AUC: area under the (Receiver Operating Characteristics) curve
CSF: cerebrospinal fluid
CNS: central nervous system
GO: gene ontology
GPI: glycolphosphatidylinositol
HPA: Human Protein Atlas
NfL: Neurofilmant light chain
PEA: proximity extension assay
PET: positron emission tomography
