# Supplementary material for: Deciphering Protein Secretion from the Brain to Cerebrospinal Fluid for Biomarker Discovery
Source: J Proteome Res. 2023 Aug 22;22(9):3068–80. doi: 10.1021/acs.jproteome.3c00366 (PMC10476268; doi:10.1021/acs.jproteome.3c00366)
Supplement: Supplementary file 1 — pr3c00366_si_001.pdf [file pr3c00366_si_001.pdf]

Supplemental Data

Deciphering protein secretion from the brain to cerebrospinal fluid for biomarker discovery

Katharina Waury[1], Renske de Wit[1], Inge M.W. Verberk[2], Charlotte E. Teunissen[2], and Sanne Abeln[1,]*

[1]Department of Computer Science, Vrije Universiteit Amsterdam, 1081 HV Amsterdam, The Netherlands

[2]Neurochemistry Laboratory, Department of Clinical Chemistry, Amsterdam Neuroscience, VU University Medical Center, Amsterdam UMC, 1081 HV Amsterdam, The Netherlands

** Corresponding author: Sanne Abeln (s.abeln@vu.nl)*

1. Supplementary information for Experimental Section
    a. CSF data set curation
    b. Feature generation
2. Supplementary tables
    a. Table S1. Included CSF proteomics studies to establish the AD CSF proteome.
    b. Table S2. Gene ontology term enrichment analysis of CSF and non-CSF brain proteins (separate file)
    c. Table S3. Predicted probability scores for full human proteome (separate file)
3. Supplementary figures
    a. Figure S1. Overlap of CSF proteomics studies in health and Alzheimer's Disease.
    b. Figure S2. Ectodomain shedding and extracellular vesicle (EV) association in CSF and non-CSF brain proteins
    c. Figure S3. Enriched motifs in CSF and non-CSF brain proteins.
    d. Figure S4. Differences in brain protein abundance and RNA tissue distribution in false positive predicted brain detected proteins
4. References

Supplementary information for Experimental Section

CSF data set curation

The data set of Macron2018A (7) was retrieved from Supporting Information Table S1. The number of peptides per unique Uniprot ID (referred to as Protein Accession Number in the table) was counted. If several Uniprot IDs were associated with a unique protein, the first Uniprot ID was kept. All unique proteins reported in the study could be identified.

The data of Macron2020 (16) was collected from Supplementary Table S1 of the publication. The same data curation steps were taken as reported for Macron2018A. All unique proteins reported in the study could be identified.

The Zhang2015 (17) study published only the unique proteins which were identified by at least two different peptides in Table 1 of the Data Article. The maximum number of identified peptides across the columns Flow-through Proteins, Original Proteins, and Bound Proteins was kept as the peptide

count for each reported protein. Because of the two-unique-peptides criterion only 2513 of the total 3256 unique proteins were included.

The results of the Guldbrandsen2014 (18) study were accessed through the CSF Proteome Resource at https://proteomics.uib.no/csf-pr-id/. The following protein data sets were downloaded: CSF GLYCO MIXEDMODE, CSF MIXED-MODE DEPLETED FRACTION, CSF MIXED-MODE BOUND FRACTION, CSF GEL DEPLETED FRACTION, CSF GEL BOUND FRACTION. For all five data sets only unique proteins with at least one associated peptide and that are validated were included. The highest reported unique peptide account across the five data sets was kept for each unique protein. The total number of unique proteins (2484) comprised 80.62% of the reported 3081 proteins identified in the study.

The data of Macron2018B (19) was collected from Supplementary Table S1 of the publication. The same data curation steps were taken as reported for Macron2018A. All unique proteins reported in the study could be identified.

The identified peptides and proteins of the Schutzer20106 (20) study were identified by IPI instead of Uniprot ID. The IPI-to-Uniprot conversion tool of the bioDBnet web server (64) at https://biodbnet-abcc.ncifcrf.gov/db/db2db.php was used to map IPI to Uniprot IDs. The number of peptides per IPI was used to derive the associated peptide count. Of the reported 2630 unique proteins 2067 (78.59%) could be matched to a known Uniprot ID.

The Higginbotham2020 (21) data was obtained from Supplementary Table S2A containing all proteins identified in their discovery cohort. Information on isoforms was not considered leading to all reported 2828 unique proteins being included.

Of the 2327 proteins identified in the Sathe2019 (22) study and listed in Supplementary Table 3, we could map 2310 proteins to a unique Uniprot ID within the human proteome.

Bader2020 (23) reported 1484 unique proteins that have been identified in at least 20 out of the 197 included samples. All of these proteins were listed in Dataset EV3. If multiple Uniprot IDs were reported for one protein, the first identifier was retained.

Feature generation

For all human proteins in our training, test and control data sets with a unique Uniprot ID the canonical protein sequence was downloaded. Subsequently, 52 sequence-based features were produced for each entry for the machine learning model to learn on.

The length of the protein sequence was derived as a feature. The Biopython Bio.SeqUtils package (65) was utilized to calculate molecular weight, the percentage of each amino acid type in the sequence, isoelectric point, and instability index (29). The amino acid type related features are represented by their one-letter code.

NetSurfP-2.0 (30) was downloaded and run locally to produce secondary structure (helix, sheet, coil) and disorder prediction for protein sequences. NetSurfP-2.0 was run using MMseqs2 and the Uniclust30 database (Release: 2017_04) to generate the required sequence profiles for prediction. The per-residue predictions of disorder and secondary structure were aggregated across the entire protein sequence to obtain an average disorder score and the proportion of each secondary structure element of the sequence.

SignalP-6.0 (31) was downloaded and run locally to predict signal peptides within the protein sequence. The slow mode was used, and the prediction was limited to only eukaryotic signal peptide prediction.

The glycosylation predictor NetNglyc-1.0 (32) was downloaded and run locally. Only high confidence glycosylation sites were included (indicated by +++). The prediction results were then implemented as a binary feature indicating presence or absence of any glycosylation site. GlycoMine (33) prediction results of C-, N-, and O-linked glycosylation sites of the entire human proteome are provided online at https://glycomine.erc.monash.edu/Lab/GlycoMine/. These data sets were downloaded and used to annotate proteins regarding the presence or absence of glycosylation sites. The thresholds reported at https://glycomine.erc.monash.edu/Lab/GlycoMine/ were used to determine a positive prediction.

DeepLoc-1.0 (34) was downloaded and run locally for subcellular localization prediction based on sequence. Every protein is assigned to the subcellular localization with the highest predicted probability out of ten possible classes: Cytoplasm, nucleus, cell membrane, extracellular, mitochondrion, endoplasmic reticulum, Golgi apparatus, peroxisome, lysosome/vacuole, and plastid.

TMHMM-2.012 (35) was used to predict transmembrane residues and regions from sequence. The web server at https://services.healthtech.dtu.dk/service.php?TMHMM-2.0 was accessed and the predicted number of transmembrane helices (PredHel), the number of transmembrane residues in the entire sequence (ExpAA) as well as in the first 60 residues (First60ExpAA) were extracted as a feature. Transmembrane regions were also included as a binary feature indicating absence or presence of any transmembrane helix.

The web server of NetGPI-1.1 (36) at https://services.healthtech.dtu.dk/service.php?NetGPI was used to produce predictions of GPI-anchors from sequence. Any protein sequences with the result "GPI-Anchored" were annotated as such.

The ScanProsite tool (38) (https://prosite.expasy.org/scanprosite/) was used to identify motifs of the PROSITE database (37) (Release 2022_01) in the brain elevated proteome. Patterns significantly enriched in either the CSF positive or negative group were implemented as a feature indicating presence or absence in the protein sequences. For more information on the included patterns, see the associated PROSITE documentation:

- EGF1 (PROSITE accession: PS00022, https://prosite.expasy.org/PDOC00021);
- EGF2 (PROSITE accession: PS01186, https://prosite.expasy.org/PDOC00021);
- Cadherin-1 (PROSITE accession: PS00232, https://prosite.expasy.org/PDOC00205);
- G-protein receptor F1 (PROSITE accession: PS00237, https://prosite.expasy.org/PDOC00210);
- Zinc Finger C2H2 (PROSITE accession: PS00028, https://prosite.expasy.org/PDOC00028);
- Homeobox (PROSITE accession: PS00027, https://prosite.expasy.org/PDOC00027).

Annotations of ectodomain shedding proteins were taken from two previously published efforts to collect all known human ectodomain shedding proteins. The known shedding proteins listed on the web server DeepSMP (40) (http://www.csbg-jlu.info/DeepSMP/browse.php19) and the human shedding proteins in the SheddomeDB (41) (https://bal.lab.nycu.edu.tw/sheddomedb/app/browse) were combined and used to annotate ectodomain shedding proteins in our protein set.

Curated annotations of EV associated proteins from a recently published study were added as a feature (27).

Supplementary tables

Table S1. Included CSF proteomics studies to establish the AD CSF proteome. AD – Alzheimer's Disease; HC – healthy controls

Study name	Reported CSF proteins	Included CSF proteins	HC:AD subject ratio	Female: male subject ratio	Median subject age in years	Ref.
Higginbotham 2020	2875	2828	20:20	9:11 (HC)<br>8:12 (AD)	N.A.	(21)
Sathe2019	2327	2310	5:5	3:2 (HC)<br>4:1 (AD)	67 (64-83) (HC)<br>73 (57-80) (AD)	(22)
Bader2020	1484	1484	109:88	50:59 (HC)<br>49:39 (AD)	68 (20-88) (HC)<br>72 (57-88) (AD)	(23)

Table S2. Gene ontology term enrichment analysis of CSF brain proteins (separate file)

Table S3. Predicted probability scores for full human proteome (separate file)

Supplementary figures

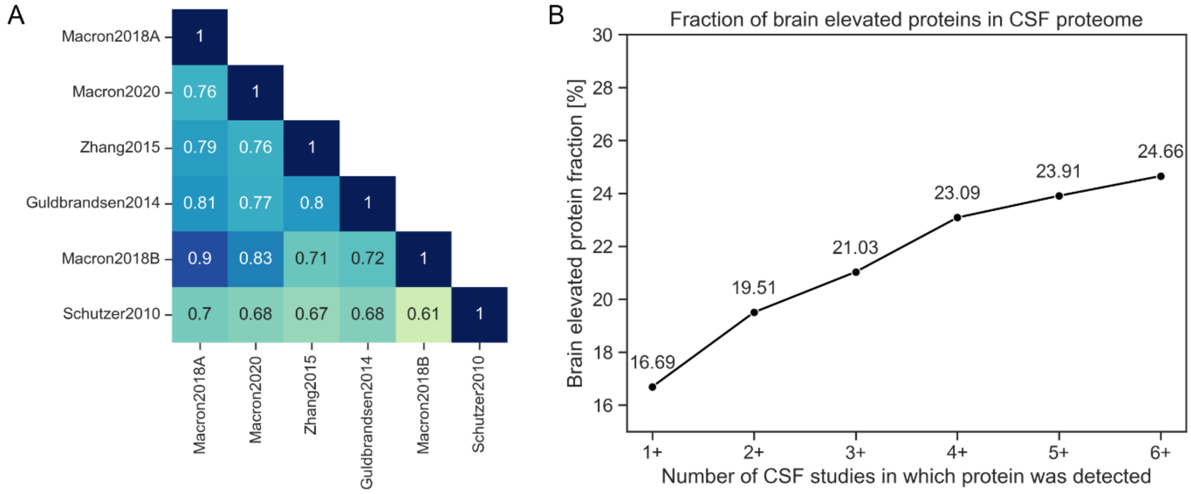

Figure S1. Overlap of CSF and brain proteome datasets. (A) The inter-study proteome variability is indicated by a heat map showing the protein overlap between the studies which are sorted by protein set size. The protein overlap is displayed as a value between 0 (no overlap) and 1 (complete overlap). Protein sets between different studies have an overlap between 0.61 and 0.9. (B) The relative overlap of the CSF proteome with the brain elevated HPA proteome increases in the higher stringency CSF protein sets.

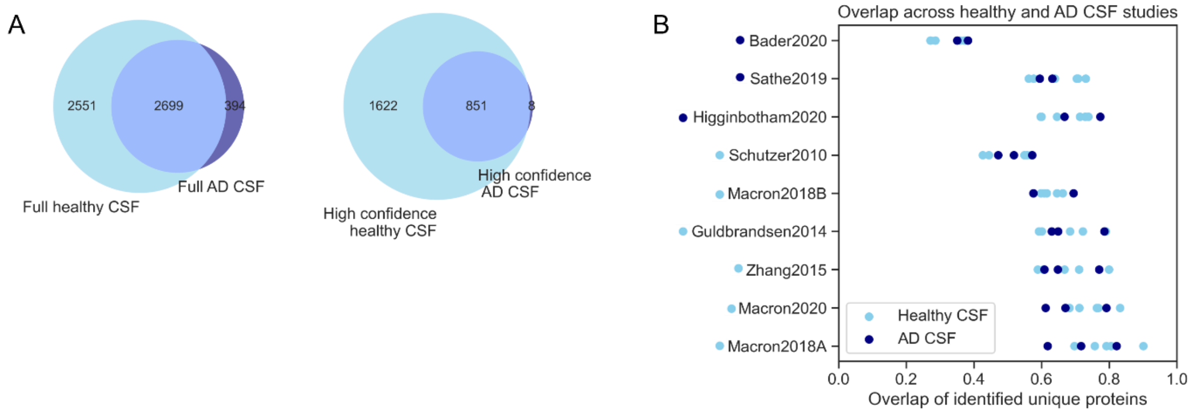

Figure S2. Overlap of proteome composition in healthy and Alzheimer's Disease CSF studies. (A) The overlap of the healthy CSF proteome with the AD CSF proteome is high and increasing in the high confidence CSF3+ protein sets. Note that only proteins that are part of the human proteome were included here. (B) The fraction of shared unique proteins between healthy and AD CSF studies was compared to investigate if disease status strongly influences CSF composition similarity between studies. Each row displays for one study the relative overlap of protein sets with the other included healthy and AD CSF studies (see **Table 1** and **Table S1**) in a pair-wise manner. The dot colour indicates if the compared study investigated healthy or AD CSF. The relative overlap of the protein sets is not systematically affected by disease status (B). AD – Alzheimer's Disease

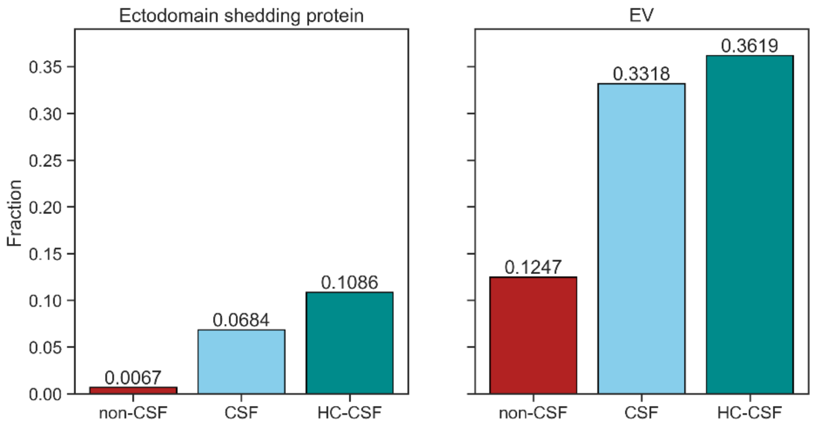

Figure S3. Ectodomain shedding and EV association in CSF and non-CSF brain proteins. CSF secreted proteins are significantly more often annotated as ectodomain shedding and EV associated proteins than non-CSF proteins. This observation is more distinct in the higher confidence CSF proteins (CSF2+, CSF3+). Both properties could explain the high occurrence of single-transmembrane proteins in the CSF (Figure 3D). EV – extracellular vesicle

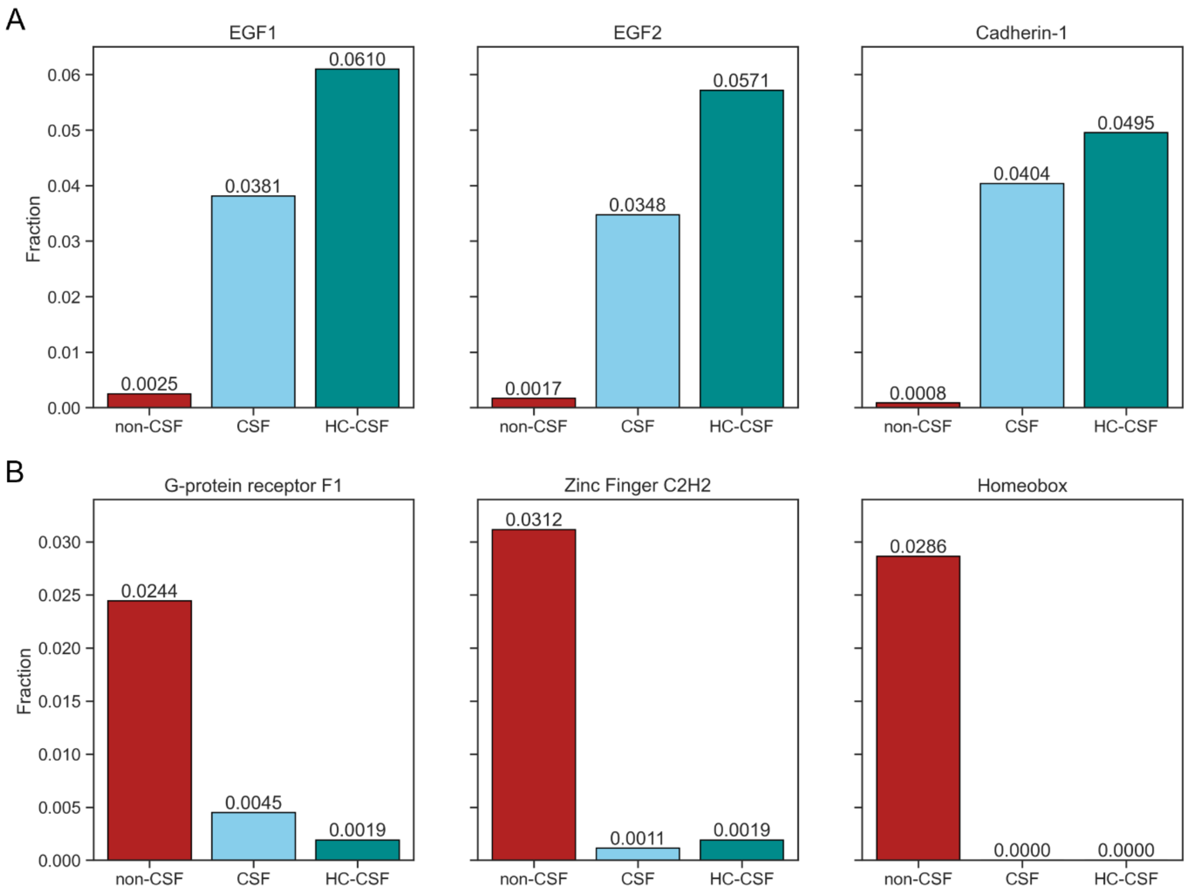

Figure S4. Enriched motifs in CSF and non-CSF brain proteins. (A) The PROSITE patterns of EGF-like domain signature 1, EGF-like domain signature 2 and Cadherin-1 are strongly enriched in CSF brain proteins. (B) Patterns of G-protein receptor F1, the Zinc Finger C2H2 and homeobox domain are predominantly found in non-CSF brain proteins. EGF – epidermal growth factor

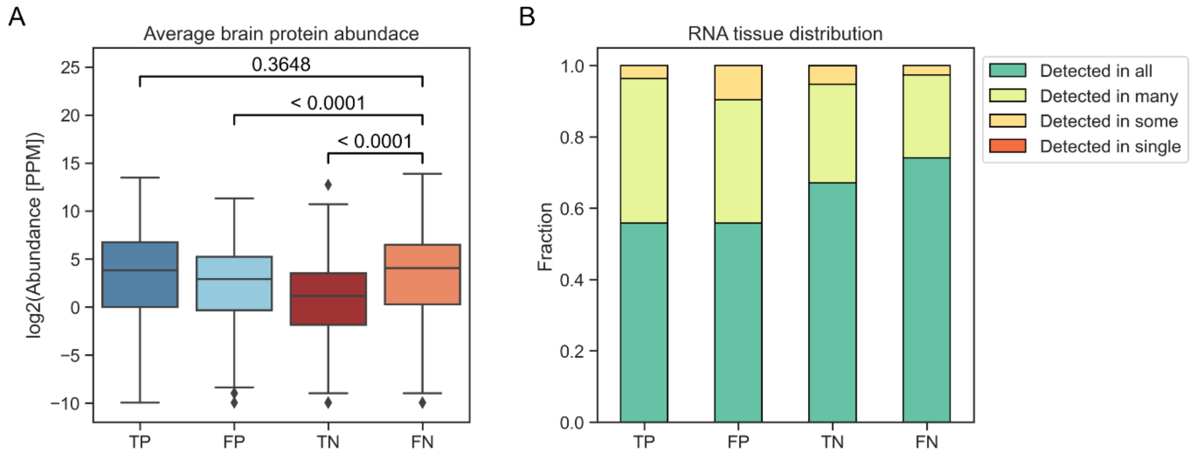

Figure S5. Differences in brain protein abundance and RNA tissue distribution in false negative predicted brain detected proteins. (A) Comparison of protein abundances shows that proteins detected in CSF but not predicted to be secreted have a much higher average abundance in the brain according to PaxDB annotations. (B) RNA tissue distribution according to the HPA reveals a higher fraction of proteins detected in all human tissues in the false negative group compared to other proteins. High abundance and ubiquitous expression might explain easy detection of these proteins in CSF. FN – false negative; FP – false positive; PPM – parts per million; TN – true negative; TP – true positive

References

(7) Macron, C.; Lane, L.; Galindo, A. N.; Dayon, L. Deep Dive on the Proteome of Human Cerebrospinal Fluid: A Valuable Data Resource for Biomarker Discovery and Missing Protein Identification. *Journal of Proteome Research* **2018**, *17* (12), 4113–4126. https://doi.org/10.1021/acs.jproteome.8b00300.

(16) Macron, C.; Lavigne, R.; Galindo, A. N.; Affolter, M.; Pineau, C.; Dayon, L. Exploration of Human Cerebrospinal Fluid: A Large Proteome Dataset Revealed by Trapped Ion Mobility Time-of-Flight Mass Spectrometry. *Data in Brief* **2020**, *31*, 105704. https://doi.org/10.1016/j.dib.2020.105704.

(17) Zhang, Y.; Guo, Z.; Zou, L.; Yang, Y.; Zhang, L.; Ji, N.; Shao, C.; Wang, Y.; Sun, W. Data for a Comprehensive Map and Functional Annotation of the Human Cerebrospinal Fluid Proteome. *Data in Brief* **2015**, *3*, 103–107. https://doi.org/10.1016/j.dib.2015.02.004.

(18) Guldbrandsen, A.; Vethe, H.; Farag, Y.; Oveland, E.; Garberg, H.; Berle, M.; Myhr, K.-M.; Opsahl, J. A.; Barsnes, H.; Berven, F. S. In-Depth Characterization of the Cerebrospinal Fluid (CSF) Proteome Displayed Through the CSF Proteome Resource (CSF-PR). *Molecular & Cellular Proteomics* **2014**, *13* (11), 3152–3163. https://doi.org/10.1074/mcp.m114.038554.

(19) Macron, C.; Lane, L.; Galindo, A. N.; Dayon, L. Identification of Missing Proteins in Normal Human Cerebrospinal Fluid. *Journal of Proteome Research* **2018**, *17* (12), 4315–4319. https://doi.org/10.1021/acs.jproteome.8b00194.

(20) Schutzer, S. E.; Liu, T.; Natelson, B. H.; Angel, T. E.; Schepmoes, A. A.; Purvine, S. O.; Hixson, K. K.; Lipton, M. S.; Camp, D. G.; Coyle, P. K.; Smith, R. D.; Bergquist, J. Establishing the Proteome of Normal Human Cerebrospinal Fluid. *PLoS ONE* **2010**, *5* (6), e10980. https://doi.org/10.1371/journal.pone.0010980.

(21) Higginbotham, L.; Ping, L.; Dammer, E. B.; Duong, D. M.; Zhou, M.; Gearing, M.; Hurst, C.; Glass, J. D.; Factor, S. A.; Johnson, E. C. B.; Hajjar, I.; Lah, J. J.; Levey, A. I.; Seyfried, N. T. Integrated Proteomics Reveals Brain-Based Cerebrospinal Fluid Biomarkers in Asymptomatic and

Symptomatic Alzheimer's Disease. *Science Advances* **2020**, *6* (43), eaaz9360. https://doi.org/10.1126/sciadv.aaz9360.
(22) Sathe, G.; Na, C. H.; Renuse, S.; Madugundu, A. K.; Albert, M.; Moghekar, A.; Pandey, A. Quantitative Proteomic Profiling of Cerebrospinal Fluid to Identify Candidate Biomarkers for Alzheimer's Disease. *Prot. Clin. Appl.* **2019**, *13* (4), 1800105. https://doi.org/10.1002/prca.201800105.
(23) Bader, J. M.; Geyer, P. E.; Müller, J. B.; Strauss, M. T.; Koch, M.; Leypoldt, F.; Koertvelyessy, P.; Bittner, D.; Schipke, C. G.; Incesoy, E. I.; Peters, O.; Deigendesch, N.; Simons, M.; Jensen, M. K.; Zetterberg, H.; Mann, M. Proteome Profiling in Cerebrospinal Fluid Reveals Novel Biomarkers of Alzheimer's Disease. *Mol Syst Biol* **2020**, *16* (6), e9356. https://doi.org/10.15252/msb.20199356.
(27) Waury, K.; Gogishvili, D.; Nieuwland, R.; Chatterjee, M.; Teunissen, C. E.; Abeln, S. *Proteome Encoded Determinants of Protein Sorting into Extracellular Vesicles*; preprint; 2023. https://doi.org/10.1101/2023.02.01.526570.
(29) Guruprasad, K.; Reddy, B. V. B.; Pandit, M. W. Correlation between Stability of a Protein and Its Dipeptide Composition: A Novel Approach for Predicting in Vivo Stability of a Protein from Its Primary Sequence. *Protein Engineering, Design and Selection* **1990**, *4* (2), 155–161. https://doi.org/10.1093/protein/4.2.155.
(30) Klausen, M. S.; Jespersen, M. C.; Nielsen, H.; Jensen, K. K.; Jurtz, V. I.; Sønderby, C. K.; Sommer, M. O. A.; Winther, O.; Nielsen, M.; Petersen, B.; Marcatili, P. NetSurfP-2.0: Improved Prediction of Protein Structural Features by Integrated Deep Learning. *Proteins: Structure, Function, and Bioinformatics* **2019**, *87* (6), 520–527. https://doi.org/10.1002/prot.25674.
(31) Teufel, F.; Armenteros, J. J. A.; Johansen, A. R.; Gíslason, M. H.; Pihl, S. I.; Tsirigos, K. D.; Winther, O.; Brunak, S.; Heijne, G. von; Nielsen, H. SignalP 6.0 Predicts All Five Types of Signal Peptides Using Protein Language Models. *Nature Biotechnology* **2022**, *40* (7), 1023–1025. https://doi.org/10.1038/s41587-021-01156-3.
(32) Gupta, R.; Brunak, S. Prediction of Glycosylation across the Human Proteome and the Correlation to Protein Function. *Pacific Symposium on Biocomputing* **2002**, *7*, 310–322.
(33) Li, F.; Li, C.; Wang, M.; Webb, G. I.; Zhang, Y.; Whisstock, J. C.; Song, J. GlycoMine: A Machine Learning-Based Approach for Predicting N-, C- and O-Linked Glycosylation in the Human Proteome. *Bioinformatics* **2015**, *31* (9), 1411–1419. https://doi.org/10.1093/bioinformatics/btu852.
(34) Almagro Armenteros, J. J.; Sønderby, C. K.; Sønderby, S. K.; Nielsen, H.; Winther, O. DeepLoc: Prediction of Protein Subcellular Localization Using Deep Learning. *Bioinformatics* **2017**, *33* (21), 3387–3395. https://doi.org/10.1093/bioinformatics/btx431.
(35) Krogh, A.; Larsson, B.; Heijne, G. von; Sonnhammer, E. L. L. Predicting Transmembrane Protein Topology with a Hidden Markov Model: Application to Complete Genomes. *Journal of Molecular Biology* **2001**, *305* (3), 567–580. https://doi.org/10.1006/jmbi.2000.4315.
(36) Gíslason, M. H.; Nielsen, H.; Armenteros, J. J. A.; Johansen, A. R. Prediction of GPI-Anchored Proteins with Pointer Neural Networks. *Current Research in Biotechnology* **2021**, *3*, 6–13. https://doi.org/10.1016/j.crbiot.2021.01.001.
(37) Sigrist, C. J. A.; Castro, E. de; Cerutti, L.; Cuche, B. A.; Hulo, N.; Bridge, A.; Bougueleret, L.; Xenarios, I. New and Continuing Developments at PROSITE. *Nucleic Acids Research* **2012**, *41* (D1), D344–D347. https://doi.org/10.1093/nar/gks1067.
(38) Castro, E. de; Sigrist, C. J. A.; Gattiker, A.; Bulliard, V.; Langendijk-Genevaux, P. S.; Gasteiger, E.; Bairoch, A.; Hulo, N. ScanProsite: Detection of PROSITE Signature Matches and ProRule-Associated Functional and Structural Residues in Proteins. *Nucleic Acids Research* **2006**, *34* (Web Server), W362–W365. https://doi.org/10.1093/nar/gkl124.
(39) Haynes, W. A.; Tomczak, A.; Khatri, P. Gene Annotation Bias Impedes Biomedical Research. *Scientific Reports* **2018**, *8* (1), 1362. https://doi.org/10.1038/s41598-018-19333-x.

(40) Cao, Z.; Du, W.; Li, G.; Cao, H. DEEPSMP: A Deep Learning Model for Predicting the Ectodomain Shedding Events of Membrane Proteins. *Journal of Bioinformatics and Computational Biology* **2020**, *18* (03), 2050017. https://doi.org/10.1142/s0219720020500171.
(41) Huang, W.-Y.; Wu, K.-P. SheddomeDB 2023: A Revision of an Ectodomain Shedding Database Based on a Comprehensive Literature Review and Online Resources. *J. Proteome Res*. 2023, acs.jproteome.3c00001. https://doi.org/10.1021/acs.jproteome.3c00001.
(64) Mudunuri, U.; Che, A.; Yi, M.; Stephens, R. M. BioDBnet: The Biological Database Network. *Bioinformatics* **2009**, *25* (4), 555–556. https://doi.org/10.1093/bioinformatics/btn654.
(65) Cock, P. J. A.; Antao, T.; Chang, J. T.; Chapman, B. A.; Cox, C. J.; Dalke, A.; Friedberg, I.; Hamelryck, T.; Kauff, F.; Wilczynski, B.; Hoon, M. J. L. de. Biopython: Freely Available Python Tools for Computational Molecular Biology and Bioinformatics. *Bioinformatics* **2009**, *25* (11), 1422–1423. https://doi.org/10.1093/bioinformatics/btp163.
